# Supplementary material for: Causal Relationship between Aging and Anorexia Nervosa: A White-Matter-Microstructure-Mediated Mendelian Randomization Analysis
Source: Biomedicines. 2024 Aug 16;12(8):1874. doi: 10.3390/biomedicines12081874 (PMC11351342; doi:10.3390/biomedicines12081874)
Supplement: Supplementary file 1 [file biomedicines-12-01874-s001.zip › Supplementary Figure/Figure S4/Singe SNP plot of MR analysis for TL to ubm-b-1505.pdf]

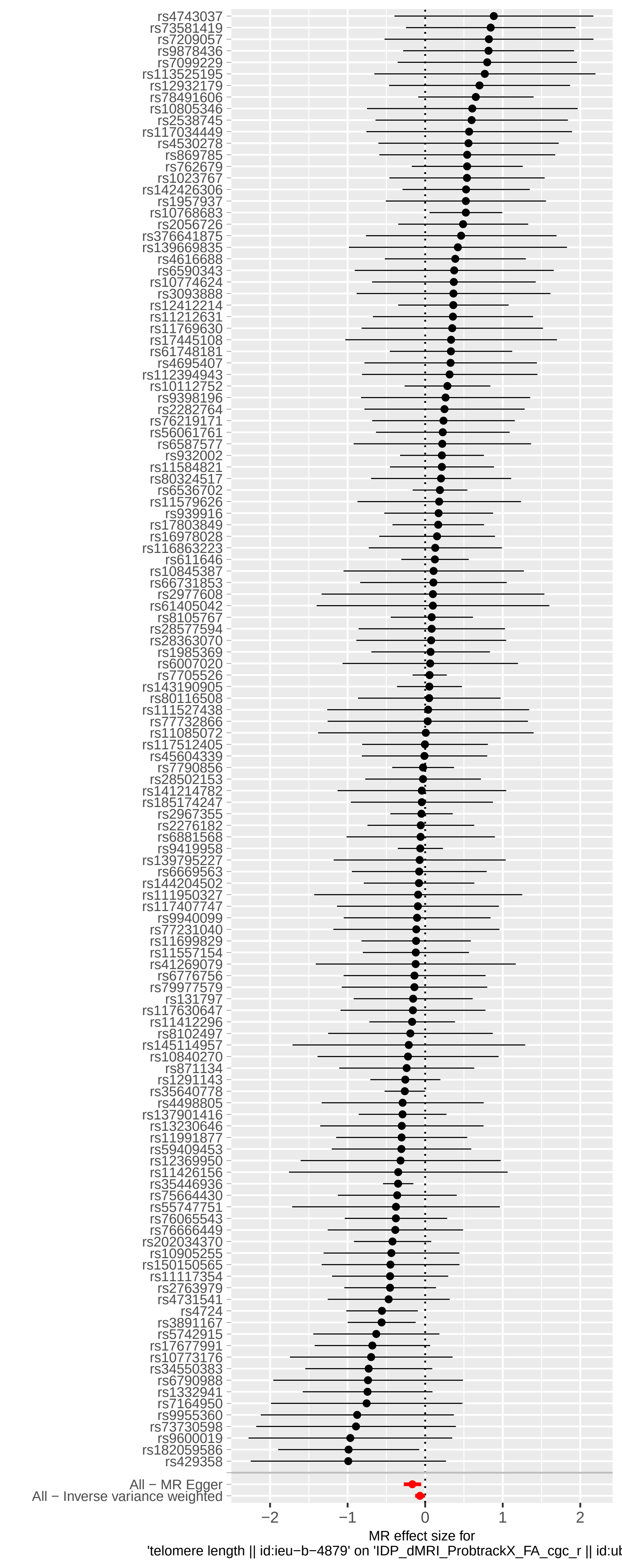
rs4743037
rs73581419
rs7209057
rs9878436
rs7099229
rs113525195
rs12932179
rs78491606
rs10805346
rs2538745
rs117034449
rs4530278
rs869785
rs762679
rs1023767
rs142426306
rs1957937
rs10768683
rs2056726
rs376641875
rs139669835
rs4616688
rs6590343
rs10774624
rs3093888
rs12412214
rs11212631
rs11769630
rs17445108
rs61748181
rs4695407
rs112394943
rs10112752
rs9398196
rs2282764
rs76219171
rs56061761
rs6587577
rs932002
rs11584821
rs80324517
rs6536702
rs11579626
rs939916
rs17803849
rs16978028
rs116863223
rs611646
rs10845387
rs66731853
rs2977608
rs61405042
rs8105767
rs28577594
rs28363070
rs1985369
rs6007020
rs7705526
rs143190905
rs80116508
rs111527438
rs77732866
rs11085072
rs117512405
rs45604339
rs7790856
rs28502153
rs141214782
rs185174247
rs2967355
rs2276182
rs6881568
rs9419958
rs139795227
rs6669563
rs144204502
rs111950327
rs117407747
rs9940099
rs77231040
rs11699829
rs11557154
rs41269079
rs6776756
rs79977579
rs131797
rs117630647
rs11412296
rs8102497
rs145114957
rs10840270
rs871134
rs1291143
rs35640778
rs4498805
rs137901416
rs13230646
rs11991877
rs59409453
rs12369950
rs11426156
rs35446936
rs75664430
rs55747751
rs76065543
rs76666449
rs202034370
rs10905255
rs150150565
rs11117354
rs2763979
rs4731541
rs4724
rs3891167
rs5742915
rs17677991
rs10773176
rs34550383
rs6790988
rs1332941
rs7164950
rs9955360
rs73730598
rs9600019
rs182059586
rs429358
All − MR Egger
All − Inverse variance weighted
−2
−1
0
1
2
MR effect size for
'telomere length || id:ieu−b−4879' on 'IDP_dMRI_ProbtrackX_FA_cgc_r || id:ub
